# Hemothorax due to a ruptured esophageal gastrointestinal stromal tumor: case report

**DOI:** 10.1186/s40792-022-01481-y

**Published:** 2022-08-11

**Authors:** Shohei Ohki, Naoki Enomoto, Daiki Kato, Shusuke Yagi, Hitomi Wake, Kyoko Nohara, Hideki Miyazaki, Toru Igari, Norihiro Kokudo, Kazuhiko Yamada

**Affiliations:** 1 Department of Surgery, Center Hospital of the National Center for Global Health and Medicine, 1-21-1, Toyama, Shinjuku, Tokyo, 162-8655 Japan; 2 Department of Pathology, Center Hospital of the National Center for Global Health and Medicine, 1-21-1, Toyama, Shinjuku, Tokyo, 162-8655 Japan

**Keywords:** Esophageal gastrointestinal stromal tumor, GIST, Hemothorax, Posterior mediastinal tumor, Tumor rupture

## Abstract

**Background:**

Esophageal gastrointestinal stromal tumors (GISTs) are rare tumors of the gastrointestinal interstitium, and comprise less than 0.7% of all GISTs. The presentation of esophageal GIST is relatively benign, commonly characterized by symptoms of dysphagia and gastrointestinal bleed. On the contrary, it is highly unusual for these tumors to present as surgical emergencies.

**Case presentation:**

Here, we describe a case of hemothorax secondary to the rupture of a massive (19 cm) esophageal GIST in a 79-year-old male. The patient presented with mild back pain, vomiting, and hypotension. A CT scan revealed significant mediastinal enlargement and left hemothorax. We conducted an emergency thoracotomy which revealed a 19 × 15 × 7 cm ruptured esophageal tumor that was bleeding profusely into the left thoracic cavity. Piecemeal resection without esophagectomy was performed to achieve hemostasis. Pathological evaluation of resected tissue confirmed the diagnosis of GIST. The patient was provided adjuvant imatinib therapy and remains progression-free at the 10-month follow-up.

**Conclusions:**

To the best of our knowledge, this is the first reported case of life-threatening hemothorax caused by a ruptured esophageal GIST. Findings from this case may aid in the diagnosis and management of these rare tumors.

## Background

Gastrointestinal stromal tumors (GISTs) are rare tumors of the gastrointestinal interstitium and esophageal GISTs are particularly unusual, accounting for only 0.7% of all GISTs [1]. Research on the diagnosis and management of esophageal GISTs is scarce and is mostly limited to case reports [2]. Common symptoms of esophageal GISTs are dysphagia and gastrointestinal bleeding [3] and it is rare for esophageal GISTs to cause significant or life-threatening hemorrhage. Very few cases have been reported [4–8], and only one case describes an esophageal GIST hemorrhaging into the thoracic cavity [9], making this an extraordinarily rare phenomenon. Surgical management, either by esophagectomy or enucleation, is indicated in esophageal GIST and obtaining a margin-negative resection is the goal [3]. Imatinib mesylate, a tyrosine kinase inhibitor, is a recent adjuvant chemotherapy agent that can significantly prolong disease-free survival in GIST [10] and its use has been reported in esophageal GISTs. Specifically, Schizas et al. reported neoadjuvant and adjuvant imatinib use in 31 patients among 105 pooled cases of esophageal GIST [11], but it is not known if adjuvant imatinib provides survival benefit in cases of incomplete resection or tumor rupture. Here, we describe a case of left hemothorax caused by ruptured esophageal GIST that was successfully managed by “piecemeal” resection without esophagectomy during an emergency thoracotomy.

## Case presentation

**Fig. 1:**
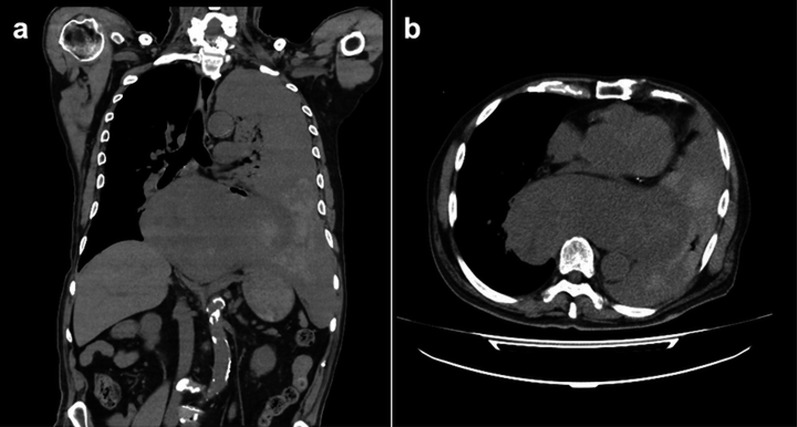
Plain computed tomography of the chest showing mediastinal enlargement and left hemothorax: **a** coronal, **b** axial

A 79-year-old male arrived by ambulance to our emergency department with chief complaints of nausea and vomiting after eating lunch. The patient was hypotensive and tachycardic on arrival and a physical exam was notable only for mild back pain and crackles in the left lower lung field. Past medical history included hypertension, type 2 diabetes mellitus, chronic kidney disease, and gastro-esophageal reflux disease which were all managed by medical therapy. As insufficient kidney function contraindicated contrast use, a non-contrast computed tomography (CT) scan was obtained (Fig. 1), which revealed massive left pleural effusion and significant mediastinal enlargement. Hence, an initial diagnosis of idiopathic esophageal rupture was made and the surgical team decided to proceed with emergency thoracotomy. The differential diagnoses considered at that time also included mediastinal hematoma, but not mediastinal mass, although the latter would have been considered if contrast CT images were available.

**Fig. 2:**
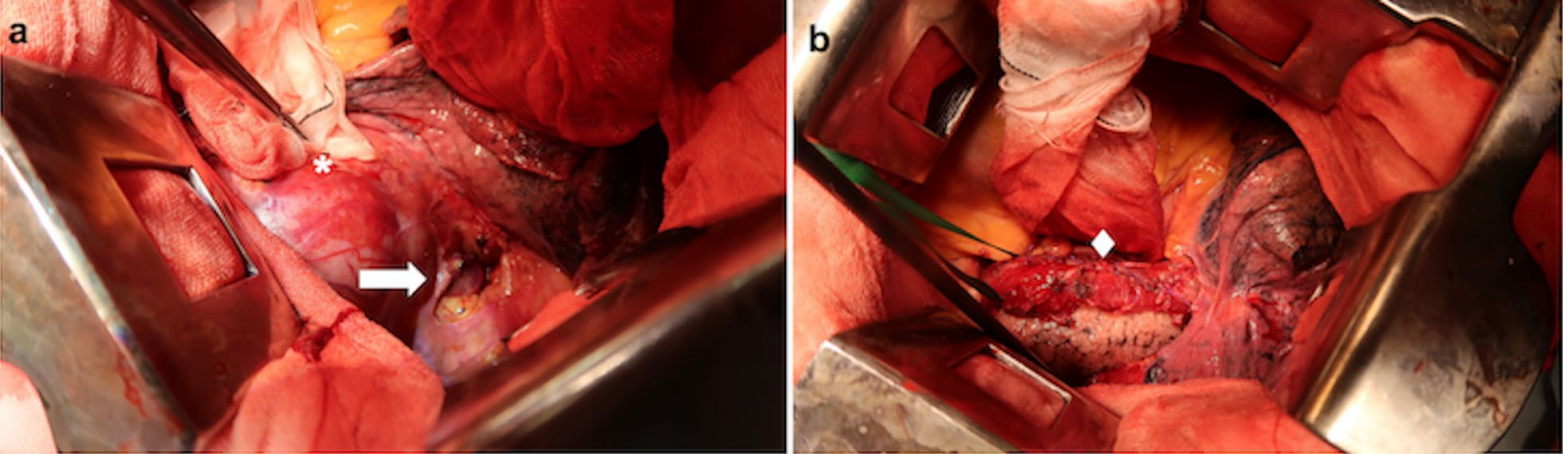
Intraoperative findings. A large posterior mediastinal tumor is visible. Gauze was applied to the point of rupture and hemorrhage (**a**) (asterisk). Mediastinal pleura incised to reveal an intact esophagus (**a**) (arrow). B After tumor resection, the esophagus was conserved and repaired with suture (**b**) (diamond)

The patient was placed in the right lateral decubitus position and the thoracotomy incision was made along the left sixth intercostal space. Independent ventilation of the right lung was established prior to thoracotomy. A massive hemothorax was noted upon entering the left thoracic cavity. After draining approximately 2 l of blood, a large tumor roughly 20 cm in size, situated caudal and posterior to the heart in the lower posterior mediastinum became visible. The tumor compressed the surrounding structures, but was encapsulated and well demarcated. Careful examination revealed that a portion of the capsule had ruptured and was hemorrhaging into the thoracic cavity (Fig. 2a), however there was no sign of esophageal rupture or perforation. Cauterization of the ruptured site was ineffective and hemorrhage was profuse, therefore resection of the tumor was deemed necessary for hemostasis. The two options for curative surgery, enucleation and esophagectomy, were both deemed impractical for the situation. In addition to the sheer difficulty of enucleating such a large tumor, the benefit was questionable since the tumor capsule had already ruptured. Esophagectomy with gastric tube reconstruction was also difficult; the first-stage esophagectomy was highly risky because of the critical condition of the patient, and the second-stage reconstructive surgery would have been complicated by significant adhesions. Therefore, we proceeded with a “piecemeal” resection of the tumor, instead of enucleation or esophagectomy, by which the tumor was resected piece by piece primarily using electrothermal and harmonic scalpels. The esophagus was subsequently liberated and its adventitia was repaired using 4-0 absorbable sutures (Fig. 2b). Damage to the lower lobe of the right lung was also repaired and chest drains were placed in the dorsal left thoracic cavity. After closing the thoracic cavity, a percutaneous gastrostomy was performed to decompress the esophagus. Finally, an enterostomy was performed to enable enteral feeding during esophageal healing. Duration of the surgery was 3 h 49 min and total blood loss was 4 l. In summary, an emergency thoracotomy was used to accomplish “piecemeal” resection of a massive mediastinal tumor with no significant surgical complications.

**Fig. 3:**
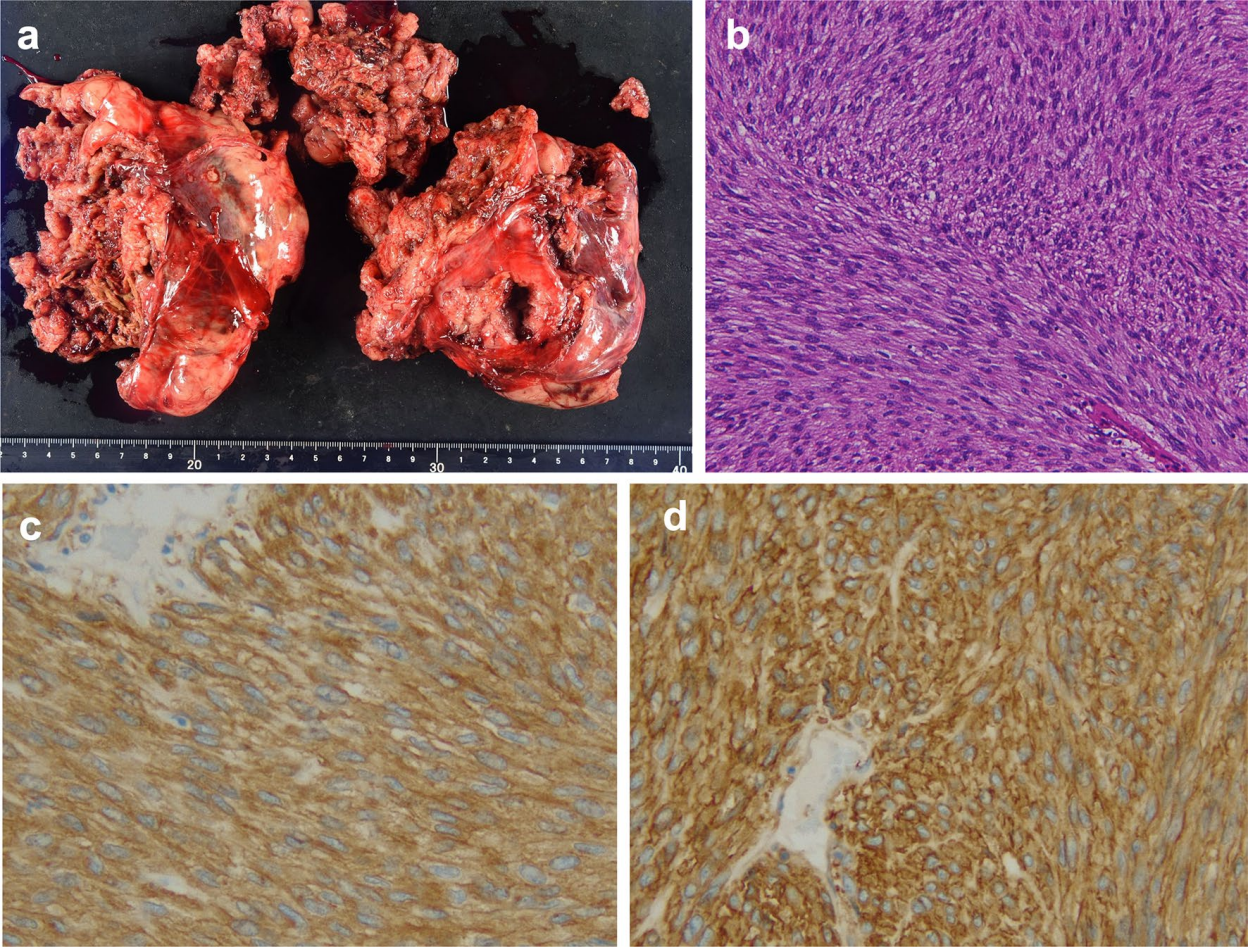
**a** Macroscopic view of the resected tumor fragments, measuring approximately 19 × 13 × 5 cm. **b** The tumor was composed of disarrayed fascicles of eosinophilic spindle cells (H&E staining ×100). By immunohistochemistry, the tumor cells were diffusely positive for c-kit (**c**) and CD34 (**d**) (×200)

Histologically, the tumor was composed of disarrayed fascicles of spindle cells with palely eosinophilic fibrillary cytoplasm (Fig. 3b). The mitotic rate was 2/50 HPFs. By immunohistochemistry, the tumor cells were diffusely positive for CD34 and c-kit (Fig. 3c, d). Desmin, S-100, AE1/AE3 and CD5 were negative. Thus, the tumor was diagnosed as an esophageal GIST of the spindle cell type. Because of the size of the tumor (> 10 cm), the patient was categorized as high-risk based on modified Fletcher classification and NCCN classifications.

**Fig. 4:**
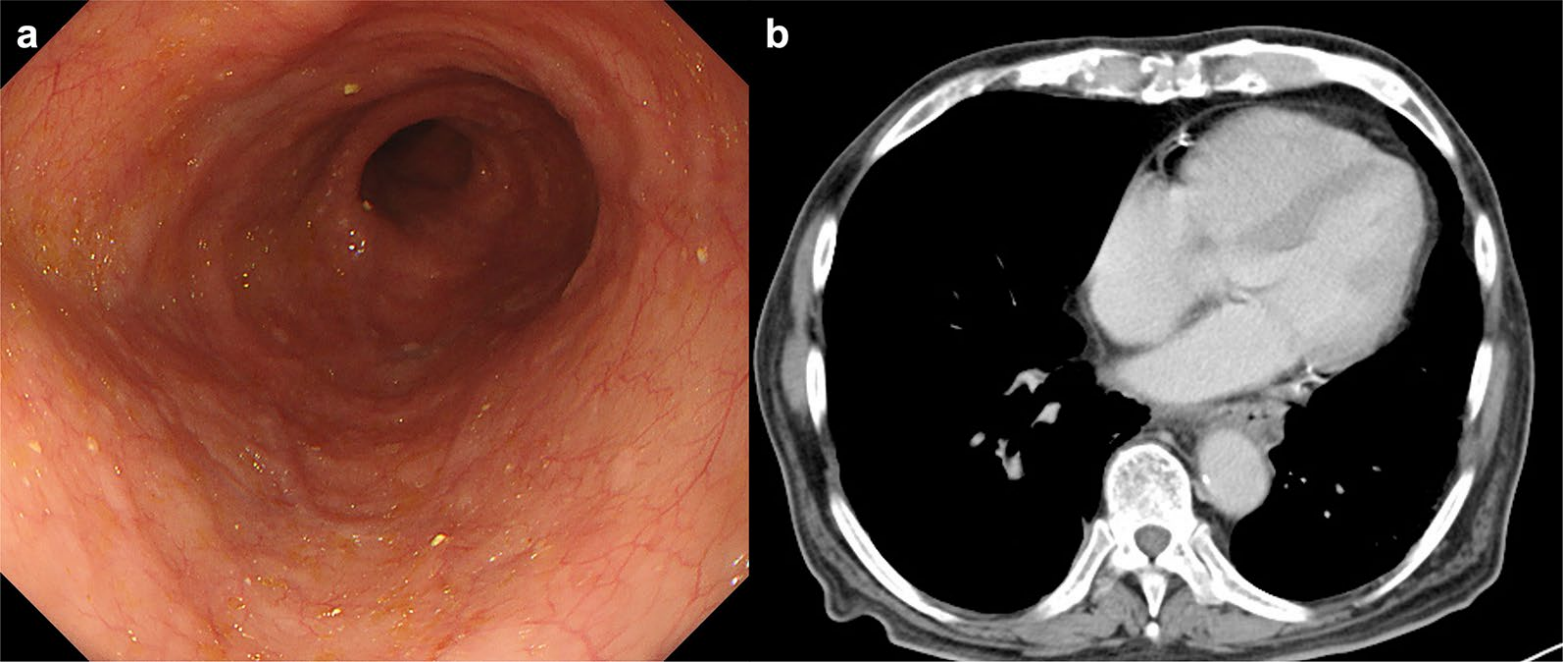
Postoperative findings. **a** Upper endoscopy image of the lower-thoracic esophagus taken 2 months post-surgery showing no sign of esophageal stricture or malignant mucosal invasion. **b** Contrast enhanced computed tomography of the chest taken 10 months post-surgery showing no sign of disease progression

The patient’s recovery from surgery was unremarkable. He was discharged on postoperative day 34. Adjuvant therapy with 400 mg imatinib mesylate daily was started as an outpatient, but discontinued after 1 month of therapy due to side-effects. The patient was offered second-line treatment with sunitinib, but declined because of his stable clinical status. Gastroscopy conducted at 2 months post-operation showed no sign of mucosal invasion or esophageal stricture (Fig. 4a). Positron emission tomography (PET) scan at 6 months, and CT scan at 10 months after surgery both demonstrated no sign of disease progression (Fig. 4b). We will continue to monitor the patient with imaging studies at least every 6 months for the foreseeable future. Restarting medical therapy will be considered if there are signs of disease progression.

## Discussion

We describe the successful surgical management of a rare case of esophageal GIST rupture. We believe this case is notable for several points. Firstly, the case is educational because the patient’s presenting symptoms closely mimicked that of esophageal perforation, a condition characterized by chest or back pain accompanied by mediastinal enlargement and pleural effusion on imaging studies. Notable deviations from the usual presentation of perforation were the lack of free air findings on imaging and the relatively mild pain experienced by the patient. In such cases, esophageal tumor rupture should be considered as a differential diagnosis.

Furthermore, an esophageal GIST rupturing into the thoracic cavity instead of the esophageal lumen is a very rare phenomenon. GISTs rupturing into the gastrointestinal lumen are not uncommon. For example, gastrointestinal bleeding due to tumor invasion is a relatively common presentation of esophageal GIST, with a reported occurrence rate of one in eight cases [12]. In stark contrast, only one case of hemothorax caused by an esophageal GIST rupturing into the thoracic cavity has ever been described in literature [9]. Interestingly, in our patient and in the previously reported case, the tumor was situated the caudal mediastinum below the tracheal bifurcation and grew in an extramural fashion. We postulate that this location allowed the tumor to grow to significant size without compressing the esophagus or surrounding structures, thus progressing unnoticed, until finally rupturing into the thoracic cavity.

Another noteworthy aspect of this case is that we conducted an emergent operation. Esophageal GISTs do not typically present as surgical emergencies, and hence, there are very few published examples of management in such situations [4–8]. One such example by Romic et al. reported an emergent “en bloc” resection of a 11 × 10 cm esophagogastric junction GIST due to uncontrollable GI bleeding after initiation of palliative imatinib therapy [7]. In our case, the profuse hemorrhage and massive size of the tumor significantly limited our options, which led to a “piecemeal”, and potentially incomplete, resection. However, the piecemeal resection was successful in controlling hemorrhage, rescuing the patient’s life, and returning him to normal daily life.

Finally, the case begs the discussion of whether incomplete resection has survival benefit in esophageal GIST. Because of the rarity of esophageal GIST, the studies to guide us on this topic do not exist. One paper that may inform us is a retrospective study of 239 patients with metastatic GIST who were on imatinib therapy and who were also provided with incomplete resection or debulking surgery of the tumor [13]. Surgery did not prolong survival in this study. As the tumor in our patient was not metastatic, we cannot confidently comment on the benefit of incomplete resection for our case. However, it is not implausible that in non-metastatic cases such as ours, even incomplete resection could have benefit by debulking the tumor. Since we had to discontinue imatinib, the prognosis of this patient is further complicated. There is evidence from the pre-imatinib era that 80% of GIST relapse occurred within 24 months of surgery [14]. Because the patient has shown no sign of disease progression at 10 months, we remain cautiously optimistic. Regardless of the lack of prognostic evidence for esophageal GIST, we believe our case is a valuable data point to guide clinicians in the future.

## Conclusions

We describe urgent surgical management of a ruptured giant esophageal GIST by emergency thoracotomy and subsequent “piecemeal” resection without esophagectomy. Although rare, ruptured esophageal GIST represents a potential differential in patients presenting with hemothorax and mediastinal enlargement.

## Data Availability

All data are presented within the manuscript.

## Abbreviations

GIST: Gastrointestinal stromal tumor
GI: Gastrointestinal
PET: Positron emission tomography
CT: Computed tomography
